# The Interrelationship of Helicase and Nuclease Domains during DNA Translocation by the Molecular Motor EcoR124I

**DOI:** 10.1016/j.jmb.2008.10.017

**Published:** 2008-12-31

**Authors:** Eva Šišáková, Marie Weiserová, Cees Dekker, Ralf Seidel, Mark D. Szczelkun

**Affiliations:** 1 Institute of Microbiology, v.v.i., Academy of Sciences of the Czech Republic, Prague, Czech Republic; 2 Kavli Institute of Nanoscience, Delft University of Technology, Lorentzweg 1, 2628 CJ Delft, The Netherlands; 3 BioTechnological Center, Dresden University of Technology, Tatzberg 47-51, D-01307 Dresden, Germany; 4 DNA–Protein Interactions Unit, Department of Biochemistry, University of Bristol, Bristol BS8 1TD, UK

**Keywords:** RM, restriction–modification, WT, wild type, TFO, triplex-forming oligonucleotide, SA, specific activity, single molecule, enzyme disorder, translocase, helicase, ATPase

## Abstract

The type I restriction–modification enzyme EcoR124I comprises three subunits with the stoichiometry HsdR_2_/HsdM_2_/HsdS_1_. The HsdR subunits are archetypical examples of the fusion between nuclease and helicase domains into a single polypeptide, a linkage that is found in a great many other DNA processing enzymes. To explore the interrelationship between these physically linked domains, we examined the DNA translocation properties of EcoR124I complexes in which the HsdR subunits had been mutated in the RecB-like nuclease motif II or III. We found that nuclease mutations can have multiple effects on DNA translocation despite being distinct from the helicase domain. In addition to reductions in DNA cleavage activity, we also observed decreased translocation and ATPase rates, different enzyme populations with different characteristic translocation rates, a tendency to stall during initiation and altered HsdR turnover dynamics. The significance of these observations to our understanding of domain interactions in molecular machines is discussed.

## Introduction

Enzyme activity can be classified by stand-alone structural domains with a particular polypeptide fold and containing specialised amino acid sequence motifs. While empirical measurements can be made using isolated domains, enzymes in nature are more typically found within larger molecular machines containing both domain fusions within a single polypeptide and domain interactions between separate polypeptides of the complex.^1^ This higher-order assembly can result in very different catalytic activities for the complete machine when compared with the domains alone.^2^ Unfortunately, the limitations of experimental measurements often lead enzymologists to take a reductionist approach, removing domains and/or subunits that may in fact impart a significant influence. Notable exceptions to this have been studies on bacterial restriction–modification (RM) enzymes.^3^ These systems protect the host cell from invasion by phage DNA and have evolved to be readily transferable:^4^ as such, they are relatively simple stand-alone machines that nonetheless have domains and features in common with more complex, but less tractable, DNA processing systems. In this study, we examined the Type I RM enzyme EcoR124I as a model system for the fusion of a nuclease domain with a helicase domain to address whether the linkage of these enzyme activities is completely passive or if there is a closer interrelationship.

**Fig. 1:**
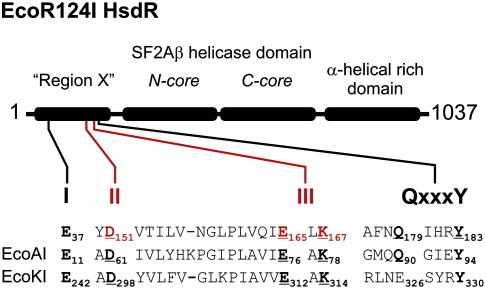
Domain structure and nuclease motifs of the Type I restriction endonucleases. EcoR124I HsdR is illustrated. Region X represents a PD-(E/D)xK superfamily nuclease fold;^7,8^ the approximate positions of the RecB-like nuclease motifs I, II, III and QxxxY are shown. The helicase domain has N- and C-core RecA-like domains characteristic of a superfamily-2 helicase;^9^ “A” indicates a motor that moves with 3′–5′ polarity; “β,” a helicase that moves on intact double-stranded DNA. The C-terminal domain is α-helix rich and is purported to be the domain that interacts with the MTase. The sequences of the EcoR124I (top), EcoAI and EcoKI nuclease motifs are shown below—conserved residues are highlighted in bold, and residues that have been mutated here (in red) and elsewhere^10,11^ are underlined. A distinct QxxxY motif is not found in EcoKI, although the tyrosine residue appears to be present.^12^ Motif I of EcoKI was identified by alignment as described previously.^8^ The primary sequence distance between motifs I and II in EcoKI is more similar to EcoAI HsdR than the longer spacing seen in EcoR124I HsdR.

The Type I RM enzyme EcoR124I comprises a complex between three protein subunits:^5,6^ HsdR, HsdM and HsdS. The HsdR subunit can be subdivided into three distinct domains (Fig. 1): an N-terminal nuclease domain (Region X) that contains motifs characteristic of a RecB family nuclease,^7,8^ a helicase domain that has the motifs and twin RecA-like folds characteristic of a superfamily-2 helicase^9^ and a C-terminal α-helical rich protein–protein interaction domain. Domain fusions are extremely common in nature; between 70% and 80% of eukaryotic proteins contain multiple domains, whereas the fraction in prokaryotes is 40%–70%.^1^ Among the helicases, nuclease chimeras are the most common: other helicase–nucleases include AddAB, Dna2 and WRN.^13–15^ We can therefore use Type I enzymes as model systems to address two general questions:

1. What effect do protein–protein contacts between subunits of a complex have on enzyme activity?
2. What effect does the presence of multiple domains within a subunit have on enzyme activity?

Studies on EcoR124I have provided answers to the first question.^16^ In isolation, the HsdR subunit has a suboptimal, non-specific ATPase activity,^17,18^ and site-specific DNA recognition and cleavage are not possible. Therefore, HsdR cannot act alone as a restriction endonuclease. To fulfil its role, it must be loaded onto the DNA by a specifically bound core methyltransferase that comprises the complex HsdM_2_HsdS_1_.^5,19^ Once loaded, the HsdR subunit has multiple enzyme activities, a more vigorous ATPase that is coupled to DNA translocation with one ATP consumed for every base pair moved,^18^ a loop translocase activity that causes movement over thousands of base pairs along the 3′–5′ strand of duplex DNA^20–23^ and a DNA cleavage activity that is typically activated upon collision with a converging HsdR motor.^5,24,25^ Thus, assembly of a complete hetero-oligomeric protein machine is required to fully activate the HsdR subunit. This assembly process may have a role in the *in vivo* control of promiscuous nuclease activity.^19^

To answer the second question on the interrelationship of connected domains and to further understand Type I RM enzymes, we wished to address whether there is any cross-talk between the nuclease and helicase activities of the HsdR polypeptide during translocation. Previous studies on the nuclease motif of the Type I RM enzyme EcoKI suggested that at least one nuclease mutation in this domain could significantly affect motor activity.^10^ To address this possibility in more depth, we made previously uncharacterised mutations of EcoR124I HsdR in motifs II and III of the RecB family nuclease (Fig. 1). The activity of these mutants was then analysed within the context of the complete RM machine using both single-molecule and bulk-solution techniques. We show that mutations in these motifs that reduced or removed DNA cleavage activity also had profound effects on motor protein activity. This suggests a close interrelationship between domains of the same polypeptide.

## Results

### Mutagenesis of motifs II and III of Region X of EcoR124I HsdR

Based on biochemical and bioinformatic analyses (Fig. 1),^7,8,10–12^ Region X of EcoR124I HsdR can be classified as part of the RecB-like nucleases of the PD-(E/D)xK superfamily.^26^ The RecB-like nucleases are defined by four amino acid motifs: I, II, III and QxxxY (Fig. 1). Similar amino acid arrangements are found in many Type I RM enzymes as well as in repair and recombination nucleases.^8^ Based on extensive studies on other PD-(E/D)xK superfamily members, the conserved charged amino acids clustered in motifs I, II and III of EcoR124I HsdR can be predicted to be central to the nuclease activity, namely in metal ion coordination and transition-state stabilisation. The QxxxY motif, which is found in many Type I enzymes, may play a role in stabilising the catalytic site near the DNA.^8^

Previous studies on Type I RM enzymes EcoAI and EcoKI have examined the roles of motifs II and III. For EcoAI, mutation of conserved residues in motif II (D61A) or motif III (E76A or K78A) (Fig. 1) produced HsdR subunits that were incapable of restricting phage growth *in vivo*, although a slow DNA nicking activity was observed with the K78A *in vitro*.^11^ The ATPase and translocase activities of the mutants were judged to be normal.^11,27^ For EcoKI, mutation of conserved residues in motif II (D298E) or motif III (E312D, E312H or K314A) (Fig. 1) produced HsdR subunits that were incapable of cleaving DNA either *in vivo* or *in vitro*.^10^ Three of the four mutants were judged to be indistinguishable from the wild type (WT) in both ATPase and translocase activities.^10,27^ However, the non-conservative mutant E312H had a significantly lower ATPase activity and no measurable translocase activity, suggesting that this mutation has a long-range effect on the helicase domain.

To extend the study on Type I nuclease mutants and their effects on translocase activity, we chose to mutate EcoR124I HsdR at the D151 residue of motif II and the E165 and K167 residues of motif III (Fig. 1). Each residue was individually substituted by a neutral alanine. To extend the collection of mutants, we also generated conservative (E165D) and non-conservative (E165H) substitutions. EcoR124I *hsdR* was mutated in the expression plasmid pACR124 (see Materials and Methods), which was used either in *in vivo* complementation assays or for expression and purification of mutant HsdR.

### *In vivo* restriction activity

To test the activity of our mutant HsdR subunits, we first analysed their restriction phenotype by testing the ability of cells expressing these mutants to restrict the growth of unmodified bacteriophage lambda. For this, we tested if the pACR124 with either WT or mutated HsdR could complement restriction in cells carrying the naturally isolated plasmid R124, which was either native [JM109(DE3)[R124], r^+^m^+^ phenotype] or chemically mutated [JM109(DE3)[R124–25], r^−^m^+^ phenotype]. R124 was chosen for complementation assays to keep the *in vivo* subunit concentrations as natural as possible. For *hsdR* expression on pACR124, only the leaky expression in the absence of IPTG was used and found to be sufficient to complement restriction.^28^ With the application of the assay to r^+^m^+^ and r^−^m^+^ cells, both DNA cleavage and EcoR124I assembly can be tested: an HsdR that is inactive in terms of DNA cleavage but that can still assemble a restriction enzyme complex would not be able to complement restriction in the r^−^m^+^ host (i.e., positive complementation) but would significantly impair restriction in the r^+^m^+^ host (i.e., negative complementation).

The complementation tests revealed that only WT *hsdR* was able to restore a restriction-proficient phenotype in the r^−^m^+^ host (Table 1); the transdominant effect of the mutant HsdR subunit on the WT HsdR, produced by the R124 plasmid, was reflected in a 1000-fold reduction in the level of EcoR124I restriction (Table 1). This indicates that the restriction deficiency of our mutants is not due to a complex assembly defect.

### *In vitro* DNA cleavage activity

For *in vitro* characterisation, complete RM enzymes were reconstituted by mixing the separately purified HsdR and MTase; reconstitution of Type I RM systems in this manner results in enzyme activity identical with that of the holoenzyme.^19,29^ All mutant HsdR subunits showed expression similar to WT HsdR. In electrophoretic mobility shift assays,^8^ we found no significant difference in the efficiency of R_2_ complex formation (i.e., HsdR_2_HsdM_2_HsdS_1_) between the WT and mutant HsdR subunits (data not shown): that is, *in vitro* assembly of the whole protein complex as monitored by specific DNA binding was not distorted by the mutations. However, it should be noted that, currently, assembly of the different species cannot be directly measured under exactly the same conditions as DNA translocation or cleavage (i.e., in the presence of ATP).

**Fig. 2:**
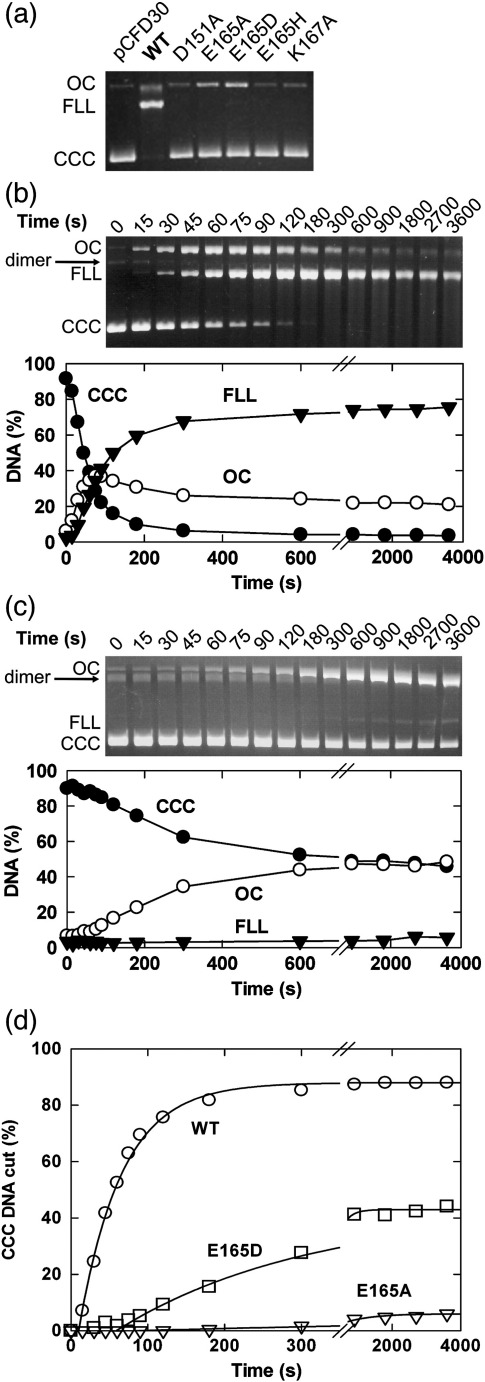
DNA cleavage activity of motif II and III nuclease mutants of EcoR124I. (a) Comparison of cleavage end points of WT and mutant HsdR subunits. Agarose gel electrophoresis was used to analyse the single-site substrate pCFD30 that was incubated for 5 min at 37 °C with saturating amounts of MTase (20 nM) and HsdR (160 nM) in the presence of 4 mM ATP (Materials and Methods). CCC is covalently closed circular DNA (substrate), OC is open circle DNA (product cut in one strand) and FLL is full-length linear DNA (product cut in both strands). (b and c) Comparison of the cleavage time course for WT HsdR (b) and E165D HsdR (c) at 25 °C. pMDS27.3 and saturating MTase (40 nM) and HsdR (100 nM) were preincubated, and the reaction was initiated by the addition of ATP to 4 mM. Samples were removed at the time points indicated and immediately quenched. The DNA substrate and products were then separated by agarose gel electrophoresis and quantified by scintillation counting (Materials and Methods). The different relative mobilities of the FLL fragments reflect different running conditions for each gel (i.e., different values in V cm^− 1^). DNA dimers are a minor contaminant in our plasmid preparations. (d) Fitting of the CCC data from WT (b), E165D (c) and E165A (data not shown) using Eq. (1) as described in Materials and Methods. The fitted parameters are given in Table 2. Under our reaction conditions, DNA cleavage by EcoR124I never goes to completion because of a background inhibition activity that competes with the translocation/cleavage process. Regardless of the observed cleavage rates, very little change in the relative levels of the species is observed beyond ∼ 5–10 min. This has been noted previously with EcoR124I using both WT enzyme and QxxxY motif mutants^8^ and has also been observed with EcoKI, but not with EcoAI (F. Peske, unpublished observations). The effect appears to be dependent on changes to the DNA that make it resistant and is not dependent on inhibition of the protein. However, the exact nature of the inhibition of DNA cleavage is unclear and is currently still under investigation (F. Peske, unpublished observations).

To test the nuclease activity of the HsdR mutants *in vitro*, we measured DNA cleavage of single-site plasmids (Materials and Methods).^8^ We first tested cleavage activity after a fixed incubation time. For the WT HsdR, maximum cleavage (as judged by the production of ∼ 80% linear DNA) can be obtained using a > 4-fold molar excess of HsdR relative to the MTase–DNA concentration and a reaction time of ∼ 5 min.^5^ An excess of HsdR is required because of the dynamic nature of the HsdR interaction with the MTase.^19^ However, to ensure that we could capture any cleavage event that occurred with our mutant enzymes, we increased the molar excess of HsdR to a > 20-fold molar excess. No cleavage activity was observed over 5 min with the D151A, E165H or K167A mutant (Fig. 2a). Increasing the HsdR concentration or the incubation time did not alter these results (data not shown). Some partial DNA nicking and the production of a small amount of full-length linear DNA were observed with the E165A and E165D mutants. These results are completely consistent with the *in vivo* results (Table 1); a DNA nicking activity is insufficient to stop phage infection.^30^

The kinetics of DNA cleavage by WT, E165A and E165D were measured over an extended timescale (Fig. 2b and c; E165A profile not shown). To compare the cleavage rates, we analysed the disappearance of the supercoiled (CCC) DNA using Eq. (1) as described in Materials and Methods (Fig. 2d; Table 2).^8^ Both E165A and E165D showed a reduction in the apparent cleavage rate and an increase in the lag offset (Table 2). These observations are consistent with a reduced DNA cleavage rate, although an increased lag can also reflect slower translocation (F. Peske, unpublished observations).^8^ The activities of these mutants—slow *in vitro* DNA nicking and a high efficiency of plating *in vivo*—are reminiscent of the slow DNA nicking activity of the positionally unrelated K78A mutant of EcoAI.^10^

### DNA translocation activity measured by single-molecule tweezers assays

**Fig. 3:**
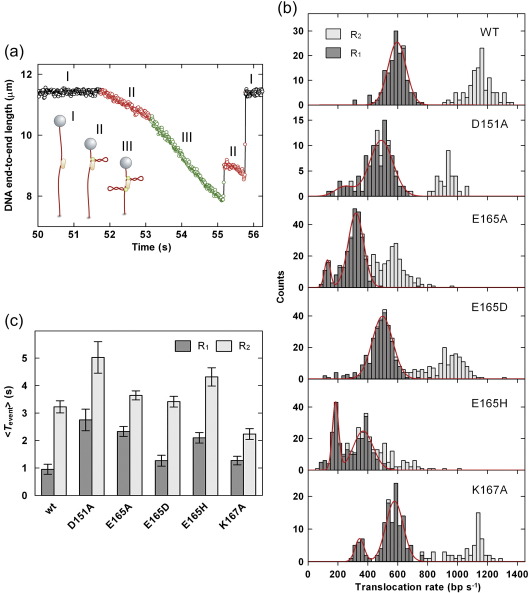
Single-molecule measurement of DNA translocation for WT and mutant HsdR subunits by magnetic tweezers. For all experiments, HsdR was at 160 nM, MTase was at 20 nM and *F* = 1.5 pN. (a) Sample single-molecule profile showing DNA length as a function of time. The inset shows a cartoon illustrating the principle of the assay, with DNA shown in red, the EcoR124I recognition site shown as a white box, the MTase shown as a light orange ovoid, the HsdR subunits shown as green circles and the magnetic bead shown in gray. See the main text for further details. The WT profile shows a period of inactivity, initiation of translocation of one HsdR subunit, initiation of translocation of a second HsdR subunit so that both motors are running simultaneously, dissociation of one subunit to leave a single translocating HsdR and, finally, dissociation of the remaining HsdR. (b) Histograms showing the translocation rates obtained from individual events binned in 25 s windows. Events are scored as either a single translocating HsdR (R_1_) or the sum of two translocating HsdR subunits (R_2_) (see the main text and Materials and Methods). Red lines are the fits of the R_1_ data to a single Gaussian (WT, E165D) or double Gaussian (D151A, E165A, E165H, K167A). Mean translocation rates for R_1_ population (from the single Gaussian fits) or both R_1_ and R_1__,slow_ populations (from the double Gaussian fits) are given in Table 2. (c) Histogram showing the average time for a translocation event (*T*_event_) when either one HsdR subunit (R_1_) or two HsdR subunits (R_2_) are translocating. Error bars represent the standard deviation of the mean. For HsdR subunits with bimodal R_1_ populations, *T*_event_ represents the average of both subpopulations.

The aforementioned results are consistent with a set of mutant enzymes in which the HsdR subunit can still bind the MTase but cannot cleave the DNA properly. Inefficient cleavage could be the result of (a) a disruption in the nuclease active site and/or (b) a disruption in the DNA translocation properties that prevents the long-range interaction of two HsdR subunits that is required to trigger cleavage. To assess if our nuclease mutants had actually affected motor activity rather than cleavage *per se*, we measured DNA translocation on a single DNA molecule using magnetic tweezers assays as described previously.^21^ Since DNA translocation by EcoR124I results in the formation of DNA loops,^22^ initiation, translocation and termination events can be visualised as characteristic sawtooth-shaped DNA shortening events (a typical profile is shown in Fig. 3a), with the slope giving the translocation rate and the duration giving the translocation lifetime. The consecutive initiation of each HsdR in an R_2_ complex can be readily visualised by a change in the slope (Fig. 3a). Therefore, the rates obtained from each event can be assigned as having arisen from either an R_1_ complex (i.e., HsdR_1_HsdM_2_HsdS_1_, with one motor running) or an R_2_ complex (both motors running), according to whether or not the slope was preceded by a clear slope of shallower gradient.

The tweezers assay can give information on initiation, translocation and termination—processes that are more difficult to measure using bulk-solution assays. Because of the long-lived DNA binding of the MTase compared with the faster, dynamic HsdR recycling,^19^ each event most likely results from a different HsdR molecule (see below), while consecutive events are likely to be the same MTase (the lifetime of the MTase being on the order of tens of minutes). For the WT and mutant HsdR subunits, we measured both translocation rates (Fig. 3b) and lifetimes (Fig. 3c).

The translocation properties of the WT enzyme were comparable with those measured previously.^18–20^ The translocation rates show symmetric distributions, with the mean R_2_ rate being almost exactly double that of the R_1_ rate (Table 2). This is because each HsdR subunit in the complex acts independently, such that the rate of two motors running is the simple sum of the rates of the individual motors.^19^ In contrast, there is a difference in the lifetime of an HsdR translocation event of approximately threefold depending on whether the HsdR is part of an R_1_ or R_2_ complex (Fig. 3c; Table 2). This difference is similar to that observed previously^19^ and is suggested to arise from a decreased stability of the HsdR–MTase interaction in an R_1_ complex compared with an R_2_ complex, possibly because of induced structural asymmetry.

Each of the HsdR mutants was then subjected to the same analysis (Fig. 3; Table 2). Overall, the results showed that mutations in motifs II and III had significant effects on the translocation rates and lifetimes. With the exception of K167A, all the mutants had reduced mean translocation rates. Moreover, the distributions of the R_1_ rates were often bimodal and/or showed significant kurtosis. This indicates that the HsdR populations not only became slower translocases but also had an increased static/dynamic disorder compared with the WT subunit. In addition, the D151A, E165A and E165H mutants showed increased translocation lifetimes, while K167A was shorter lived. The data for each mutant are subsequently described.

#### D151A

Mutation of D151 to an alanine slightly reduced the mean translocation rate for R_2_ events to 943 bp s^− 1^ (Fig. 3b). The R_1_ translocation rates were also reduced, but, strikingly, the rates showed a distinct bimodal distribution, with mean translocation rates of 243 and 490 bp s^− 1^ for the slower and faster populations, respectively (Fig. 3b; Table 2). There were also occasional R_2_ events that were significantly slower than the population mean. This indicates that this mutant comprises distinct subpopulations of HsdR subunits with distinct translocation properties. While R_1_ events were still shorter lived than R_2_ events, the overall lifetimes were longer than observed with the WT enzyme (Fig. 3c; Table 2).

#### E165A

Mutation of E165 to an alanine showed even more extreme effects, with a reduction in the R_2_ translocation rate to 565 bp s^− 1^ and an increased kurtosis in the distribution (Fig. 3b). As with the D151A mutant, a bimodal distribution of R_1_ rates was observed centred at 130 and 320 bp s^− 1^ (Table 2). The increased kurtosis in the R_2_ rates is likely due to the fact that the R_2_ events can arise from the combination of different populations: two slow HsdR subunits or two fast HsdR subunits or one fast HsdR subunit and one slow HsdR subunit. The translocation lifetime of the R_2_ complexes was similar to WT, whereas the R_1_ complexes showed more than twofold enhanced stability (Fig. 3c; Table 2).

#### E165D

Conservative mutation of E165 to aspartate produced reductions in the translocation rates for the R_1_ and R_2_ complexes to 498 and 961 bp s^− 1^, respectively (Fig. 3b; Table 2). While not clearly bimodal, the R_1_ translocation distribution shows a negative skew that probably accounts for the kurtosis in the R_2_ distribution. The translocation lifetimes of both species were similar to WT (Fig. 3c; Table 2). These data are the most similar to WT and are compatible with the retention of some nuclease activity (Fig. 2).

#### E165H

Non-conservative mutation of E165 to histidine produced a significant effect, with a clear bimodal distribution of R_1_ rates (means at 184 and 370 bp s^− 1^) and an almost-random distribution of R_2_ rates (Fig. 3b; Table 2). It was more difficult to confidently assign R_2_ events because of the different subpopulations of R_1_ species. The rates observed were similar to those seen with the E165A mutation. As in that case, the reduction in translocation rate was matched by an increase in the event durations for both R_1_ and R_2_ events (Fig. 3c; Table 2). Overall, this mutation had the most deleterious effects on translocation of all those examined. We note that the same mutation at the functionally equivalent position in EcoKI (E312H) produced an enzyme that, while still able to bind DNA, actually had no measurable translocase activity at all.^10,27^

#### K167A

Mutation of K167 to an alanine also produced a bimodal population of R_1_ species (means at 345 and 577 bp s^− 1^), albeit with the faster population equivalent to the rate seen with WT HsdR (Fig. 3b). The mean R_2_ rate was also equivalent to WT (1139 bp s^− 1^), although the distribution showed considerable kurtosis consistent with the bimodal R_1_ rates. The translocation lifetime for the R_1_ events was moderately increased, while the lifetime for the R_2_ events was moderately decreased (Fig. 3c; Table 2).

### DNA translocation activity measured by bulk-solution triplex assay

**Fig. 4:**
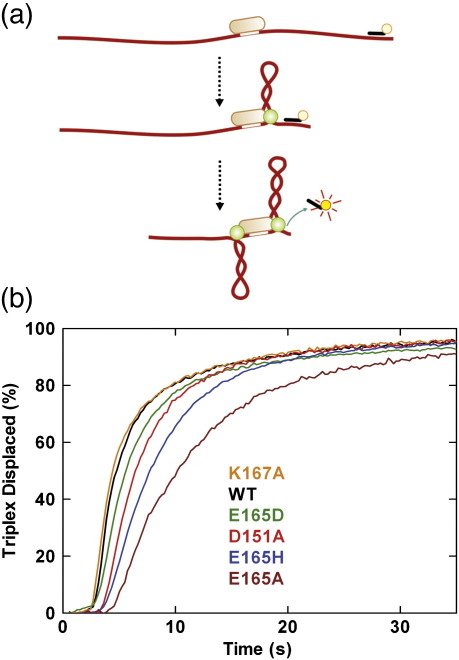
Bulk-solution measurement of DNA translocation by WT and mutant HsdR subunits by triplex displacement. For all experiments, HsdR was at 120 nM and MTase was at 30 nM. (a) Cartoon illustrating the principle of the assay, with DNA shown in red, the EcoR124I recognition site shown as a white box, the MTase shown as a light orange ovoid, the HsdR subunits shown as green circles and the TFO shown in black. See the main text for further details. (b) Triplex displacement profiles measured with a stopped-flow fluorimeter using a TAMRA-labelled TFO (0.5 nM) and a linear DNA (1 nM) with a 1517-bp spacing between the EcoR124I and triplex binding sites (Materials and Methods). Translocation rates were calculated using three further intersite spacings (data not shown) and are given in Table 2.

An alternative method for measuring DNA translocation in bulk solution is the triplex displacement assay (Fig. 4a; Materials and Methods).^20,27^ The principle of this assay is that fluorescent-labelled DNA triplex-forming oligonucleotides (TFOs) are bound downstream of an EcoR124I recognition sequence to form a triplex. HsdR translocation and collision with the triplexes cause displacement of the TFOs, which can be measured by a change in fluorescence. Accurate initiation of translocation is achieved by mixing protein–DNA solutions with ATP in a stopped-flow fluorimeter. Sample triplex displacement profiles at a spacing of 1517 bp on linear DNA are shown for WT HsdR and each mutant in Fig. 4b. Translocation rates were determined using multiple triplex spacings (data not shown)^20,27^ and are reported in Table 2. The HsdR concentration used in the assay is saturating, and the rates reported from the assay are for the translocation of a single HsdR in an R_2_ complex (i.e., equivalent to the R_1_ rate or half the R_2_ rate in the tweezers assay).

From the data obtained in the tweezers assay, the relative rates of the different HsdR subunits can be ordered as (from fastest to slowest R_1_ rates): WT ≡ K167A > E165D ≡ D151A > E165H > E165A. The order observed from the triplex assays is similar (Table 2): E165D ≡ K167A ≡ WT > D151A ≡ E165H > E165A. However, while we have previously found very good correspondence between the translocation rates determined from bulk-solution and single-molecule assays,^18^ it was striking that here none of the rates determined from the triplex assay was close to those obtained from the tweezers assays, with the former being greater than the latter in all cases. The most likely reason for the observed disparities is the increased kinetic disorder in the mutant HsdR populations (Fig. 3b). Analysis of the triplex data, as with any ensemble kinetic scheme, relies on the assumption that every enzyme has approximately the same kinetic properties (i.e., with a Boltzmann distribution).^27^ The lag phase of the triplex assay will report on the population of enzymes that arrived there the quickest. Therefore, a bimodal distribution will produce a lag that corresponds to the fastest subset only, with the slower enzymes contributing to the exponential phase of the reaction. In addition, a broader distribution of rates (as seen with the mutants in Fig. 3b) will result in apparent reductions in the exponential phase of the triplex displacement—this can actually be observed in the kinetic traces for at least some of the mutants (Fig. 4b). Given the complexity of the enzyme distributions revealed by the tweezers assay, it was not feasible to construct full kinetic schemes, as previously reported,^19^ that could accurately interpret the triplex displacement traces obtained.

### Stalling during initiation observed in single-molecule assays

**Fig. 5:**
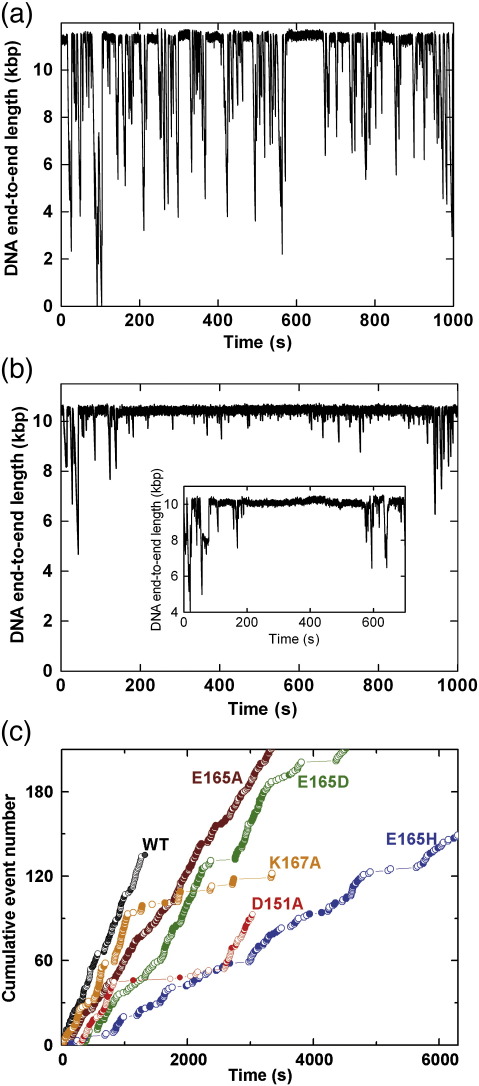
Single-molecule measurement of stalling during the initiation of DNA translocation. (a) Sample single-molecule profile for WT HsdR. (b) Sample single-molecule profile for E165D. An unusually long-lived period with decreased motor activity is observed. The inset shows a period of inactivity observed with E165D, during which no motor activity is observed at all. In all examples observed, periods of inactivity were reversible. (c) Cumulative event number *versus* time of occurrence of the event for WT and each of the mutant HsdR subunits. The cumulative event number is obtained from time traces as in (a) and (b) by scoring every single translocation event consecutively as it occurs.^19^ Long-lived periods of inactivity can be observed for the mutant HsdR subunits.

As well as measuring the translocation rate and lifetime from the tweezers assay, one can also measure the exact time of initiation of an R_1_ or R_2_ event, and these can be scored additively to give a measure of HsdR activity as a function of time (Fig. 5). While recording the traces for the mutant HsdR, noticeable regions of low activity or even inactivity were observed, generally lasting for hundreds of seconds (e.g., data for E165D in Fig. 5b). These regions are readily visualised in the accumulative event traces in Fig. 5c. For the WT enzyme (Fig. 5a), pausing can last tens of seconds but never on the same timescale as for the mutants. The distribution of times between initiation events is a compound of the second-order HsdR binding rate (to the DNA-bound MTase) and the first-order initiation rate.^19^ The longer-lived periods of inactivity/less frequent activity seen with the mutants do not fit into the expected distribution of waiting times. There are two possible causes of longer-lived periods of inactivity: (1) a conformational change in the MTase may prevent HsdR binding. However, this seems less likely as we do not regularly see such events for the WT enzyme, or (2) the association of an HsdR that is inactive. The period of inactivity may then represent the MTase binding lifetime of these HsdRs before they dissociate. Alternatively, they may represent a very slow initiation step. In some cases, there is no activity, which may be because two inactive species are bound or because the inactive HsdR prevents binding or initiation of a second HsdR (Fig. 5b, inset). In other cases, some short-lived events are seen (Fig. 5b). This may be because the same slowly initiating HsdR remains bound to the MTase for several hundreds of seconds before dissociating.

### Alterations in the dynamics of HsdR turnover

**Fig. 6:**
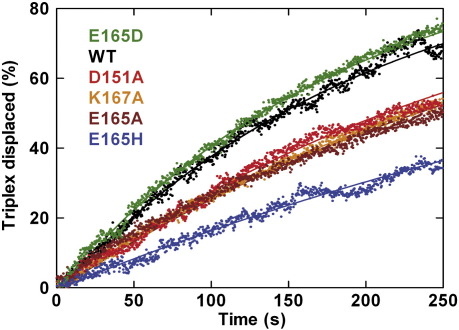
Bulk-solution measurement of DNA translocation and HsdR turnover by WT and mutant HsdR subunits using triplex displacement.^19^ Triplex displacement profiles were derived from stopped-flow experiments at a subsaturating HsdR concentration. Reactions were initiated by mixing preincubated triplex DNA (2093 bp between the EcoR124I and triplex binding sites), MTase and HsdR with an equal volume of reaction buffer plus ATP. The final solution contains 40 nM MTase, 1 nM HsdR, 5 nM DNA, 2.5 nM triplex and 4 mM ATP. Note that only 25% of the translocation events can lead to triplex displacement since there are 10 nM HsdR binding sites available (2 per DNA-bound MTase) but only 2.5 nM TFO bound.^19^ Experimental data are shown as scatter points, and continuous lines are fits to a single exponential.

Since for each of the mutants there is the possibility of (1) stalling during initiation, (2) reduced translocation rates and (3) altered translocation lifetimes (Figs. 3 and 5; Table 2), it is likely that the dynamics of HsdR turnover is also altered. We tested this using the triplex displacement assay under conditions in which HsdR is subsaturating compared with the concentration of MTase–DNA complexes (Fig. 6).^19^ To get complete triplex displacement under these conditions, each HsdR must encounter, translocate and dissociate from multiple MTase–DNA complexes. The observed rate of triplex displacement then reflects the kinetics of each of these processes. Sample profiles are shown for each of the mutants on a linear DNA with a 2093-bp spacing between the triplex and EcoR124I recognition site. With the exception of E165D, all the mutants showed a slower displacement than the WT; consistent with the other assays, the E165H mutant was the slowest, while D151A, E165A and K167A were all similar.

### ATPase activity

Our results show that mutations in motif II or III produce an enzyme population that includes species that translocate more slowly than expected and/or stall during initiation. We have recently shown that WT EcoR124I uses approximately one ATP per base pair during translocation.^18^ The observed ensemble ATPase rate is then related to the rate of translocation and the concentration of translocating species (which is only ∼ 90% populated at a steady state for WT EcoR124I due to terminated HsdR subunits that are in the act of reinitiating translocation). What is the effect of our mutants on the ATPase rates? The slower translocation and/or the stalling during initiation (Fig. 3) may be accompanied by uncoupled ATPase activity—in this case, the ATPase rate of the mutants will be similar to, or possibly more than, the WT. Alternatively, if the subunits remain tightly coupled, then stalling or slower translocation will result in less ATP usage and a slower apparent ATPase rate. To measure the ATPase activity, we utilised the coumarin-labelled phosphate binding protein sensor developed by Martin Webb,^31^ which we have used previously to extensively characterise EcoR124I.^18^ Each of the mutants showed Michaelis–Menten kinetics (data not shown). The *K*_m_ values were all similar (Table 2).^18^ More strikingly, for each mutant, the apparent maximum steady-state ATPase rate was significantly lower than that for WT. Moreover, there was no clear relationship between the translocation rates and ATPase rates. The simple explanation for these observations is that the broader distribution of translocation rates, the bimodal population distributions, the stalling events during initiation and the altered HsdR reinitiation dynamics all lead to a lower apparent ATPase rate. Consistent with all the aforementioned results, the lowest measured rate was with E165H—the equivalent EcoKI mutant, E312H, also had a very low apparent ATPase activity.^10^

## Discussion

### Central role of motifs II and III in DNA hydrolysis by Type I enzymes

We describe the mutagenesis of motifs II and III of the PD-(E/D)xK nuclease domain of the HsdR subunit of EcoR124I. We observed a significant effect on the DNA cleavage activity of these mutants (Fig. 2; Table 2), consistent with previous studies on EcoAI and EcoKI (Refs. 10,11 and 27) and as predicted by numerous studies on Type II RM enzymes and other related nucleases. However, we also observed significant effects on the ATPase and DNA translocase properties of all the mutant HsdR subunits (Figs. 3–6; Table 2). Since DNA cleavage by Type I enzymes relies on translocation, it is possible that altered translocation properties will also affect DNA cleavage. For example, the significantly slower translocation of the E165H mutant, in combination with the increased disorder and less populated R_2_ events, may lead to less successful collisions between pairs of HsdR and, in turn, less/slower cleavage. However, we would argue that all the HsdR subunits still have measurable translocation, with, in some cases, enhanced processivity. Therefore, the chance of reaching a successful collision is still high and the significant effects on cleavage are more likely due to disruptions of the active-site coordination consistent with the predicted roles of these residues.

### Disorder in DNA translocation activity produced by Region X mutations

With the advent of single-molecule measurements of enzyme kinetics, any “cosy” assumptions about protein homogeneity have been shattered: significant variations in kinetic rates have been observed both between protein molecules (static disorder) and during the reaction trajectory of a single protein molecule (dynamic disorder). An example of static disorder is the DNA unwinding activity of RecBCD, which has been shown to vary by 1.4- to 5-fold, although this observation is not necessarily consistent with all bulk-solution observations.^32,33^ An example of dynamic disorder has been observed for λ exonuclease, where temporal fluctuations over timescales similar to the time constants for the enzymatic rates were observed.^34^ In the case of EcoR124I, single-molecule measurements have actually revealed that the WT enzyme population has a relatively narrow distribution of translocation rates, as also seen here using completely different enzyme preparations.^21^ This most likely explains the good correspondence between single-molecule and ensemble-averaged values observed with WT HsdR. However, the mutations we studied here have clearly caused increased heterogeneity in the enzyme population and additional effects not seen in the WT protein. Accordingly, the ensemble-averaged ATPase and translocase rates no longer provide an accurate measure of activity. But are the disorder effects static or dynamic in nature? The observation that each EcoR124I motor event is initiated by a new HsdR recruited from solution^19^ suggests the presence of static populations since no velocity change was found during translocation events.

The Type I RM enzymes have been mooted as potential tools for the assembly of “nano-machines.”^16^ One additional goal of these studies might have been to provide a “safe” molecular motor, one that would not cut its own DNA track during translocation. What our studies also reveal is that disconnecting different enzyme activities is not trivial. Mutations of other nuclease residues (i.e., in motif I or QxxxY^8^) may be better suited to the aims of synthetic biologists. As it turns out, the WT Type I enzymes are well suited in this regard anyway, as they rarely cut linear DNA when only one recognition site is present.^21,24^

### Interpreting the interrelationship between catalytic domains in HsdR

The results presented here show that mutations in the nuclease domain of the HsdR subunit can have multiple effects on the distant helicase domain, namely, reductions in the apparent ATP rates, the appearance of increased heterogeneity and/or bimodal enzyme populations, reductions in the translocation rate, decreases in the off rate, stalling of enzymes during initiation and slower HsdR turnover. In combination, these are significant changes to the enzyme. Previous studies on Region X mutants in EcoKI and EcoAI used ensemble techniques to monitor translocation,^10,11,27^ where, as seen here (viz. the K167A mutant), it is more difficult to resolve rate and stalling heterogeneity and enzymes with abnormal population distributions can have normal-looking behaviour. Nonetheless, it should be noted that the E312H mutant of EcoKI (equivalent to the E165H mutant of EcoR124I) showed drastic reductions in its ATPase activity and no measurable translocase activity.^10,27^ Moreover, alterations in the efficiency and location of DNA cleavage observed with EcoAI nuclease mutants^11^ are also consistent with reduced translocation efficiency/increased heterogeneity.

As the helicase domain of HsdR contains all the motifs predicted to be required for mechanochemical coupling,^9^ we would not expect mutations in region X to completely ablate DNA translocation activity. On the other hand, the effects are not consistent with the endonuclease and helicase domains being completely autonomous entities. We therefore discuss two possible reasons for why changes to the nuclease domain may affect the distant helicase domain: (1) changes in overall protein fold/conformation brought about by disruptions in the packing of residues in the nuclease catalytic pocket or (2) direct interactions of the nuclease domains with the helicase/DNA during translocation.

The first, and more simple, explanation for our observations is that the mutations we introduced had a detrimental effect on the conformation of the HsdR subunit. This cannot be a complete unfolding of the polypeptide as the *in vivo* complementation assays show that the mutants can compete with WT HsdR for binding to the MTase—this would seem unlikely if the HsdR was not at least partially folded. Similarly, the translocation-inactive EcoKI nuclease mutant E312H was still able to bind DNA with an affinity equivalent to that of WT.^10^ However, it is possible to imagine changes in conformational dynamics that would still allow MTase binding without allowing DNA translocation. We certainly observed occasional long-lived stalling events that might be consistent with such an inactive population. However, we also observed a large proportion of translocation events, albeit with increased rate heterogeneity and altered properties. It may be the case that the conformations of the mutant HsdR still allow translocation but are more dynamic, resulting in greater heterogeneity in rates and occasional lapses into inactivity. Following protein purification, an enzymologist will often judge the purity of the resulting preparation by calculating a “specific activity” (SA), defined as the amount of active protein as a fraction of the total protein. A classic view is that when the protein is judged to be pure by gel electrophoresis, any reduction in SA measured using an enzyme assay could be due to an “inactive” population of molecules. We certainly saw events that could be attributed to such species in our studies. However, our results also show that changes in SA could arise because of changes in heterogeneity; this will only be revealed clearly by single-molecule approaches.

The second explanation for our observations is that there is a close mechanistic interrelationship between the nuclease and helicase domains during motion on DNA. While many isolated helicase domains still retain motor activity, full activity usually requires the cooperation of other factors (to load the motor, increase processivity, etc.).^2^ Therefore, it may be the case that EcoR124I translocation relies on protein–protein and/or protein–DNA interactions by the nuclease domain that are disrupted by Region X mutations. For example, we have previously shown that EcoR124I translocates along the 3′–5′ strand of intact double-stranded DNA but that interactions with the 5′-3′ strand are also important in maintaining processivity.^23^ The identity of the proposed “processivity clamp” is not known, but its role could be played by the nuclease domain. If mutations within the processivity clamp were to cause it to grip the DNA more tightly, then possible outcomes would be a slowing of the translocation rate (forward steps would fail against an internal roadblock) and/or an increase in processivity (dissociation of the motor would be prevented by the enhanced DNA binding). This is exactly what was observed in the majority of cases with our mutants. It has been noted previously with PD-(E/D)xK motif mutants of Type II RM enzymes that a reduction in charge local to the DNA can increase DNA affinity.^35^ This could at least explain the observations with the alanine substitutions at D151 and E165 (Fig. 3). One evolutionary advantage therefore of fusing nuclease and helicase domains might be that the resulting single polypeptide is more than the sum of its parts—the helicase motor activity can be improved with the help of the adjoining nuclease.

## Materials and Methods

### Bacterial strains, plasmids and microbiological techniques

Phage buffer, complex LB medium and *in vivo* restriction and modification assays were as described previously.^36^ The solid medium used was LB medium plus 1.5% (w/v) agar; the soft agar overlay used was LB medium plus 0.6% (w/v) agar. Antibiotics were used at the following concentrations: 100 μg ml^− 1^ of ampicillin, 50 μg ml^− 1^ of chloramphenicol and 12.5 μg ml^− 1^ of tetracycline. For the complementation assay, the IncFIV plasmid R124 carrying the EcoR124I *hsd* genes^37^ and its derivative R124–25, which carries a mutation in the *hsdR* gene (see below), were introduced into JM109(DE3) by conjugation. The virulent mutant of phage λ was used for testing restriction efficiency.^38^ Complementation assays were carried out in JM109(DE3) in the absence of IPTG (the leaky background level of HsdR expression has been found to be sufficient for restriction activity *in vivo*).^5^ The strain C122 (prototroph, Δhsd, British Culture Collection strain no. 122) or C122 plus R124 plasmids served for *in vivo* modification assays. The modification specificity of R124 was estimated as the efficiency of plating of phage λ produced by the tested strain and measured on *Escherichia coli* C122[R124] and C122 indicator strains.

### HsdR mutagenesis and protein purification

For the *in vitro* assays, the MTase and the WT and mutant derivatives of HsdR were overproduced from plasmids pJS4M^39^ and pACR124,^17^ respectively, and purified from *E. coli* JM109(DE3) using approaches described previously.^5,40^ Reconstitution of WT and mutant endonucleases was produced by mixing of the purified HsdR subunit and MTase in appropriate assay reaction buffers. HsdR mutants were generated from pACR124 with QuikChange XL site-directed mutagenesis (Stratagene) using the following oligodeoxynucleotide primers, where the mutated residues are in bold and the mutated codon is underlined (only the top strand is shown; the bottom strand was the complement sequence): 5′-CACGCTAACCGTTATG**C**CGTAACTATCCTGGTTAATGG-3′ for D151A; 5′-CCGCTGGTACAAATCG**C**ACTGAAAAAACGCGGC-3′ for E165A; 5′-CCGCTGGTACAAATCGA**C**CTGAAAAAACGCGGC-3′ for E165D; 5′-CCGCTGGTACAAATC**C**A**T**CTGAAAAAACGCGGC-3′ for E165H; and 5′-GGTACAAATCGAACTG**GC**AAAACGCGGCGTGGC-3′ for K167A. All mutants were fully sequenced and then expressed and purified as for the WT enzyme.

For the *in vivo* assays, plasmid R124–25 was prepared by chemical mutagenesis as described for EcoKI.^36^ The bacterial strain used in our experiments was *E. coli* QR47-3Δ(*hsdR-S*) (a derivative of *E. coli* QR47)^41^ carrying the plasmid R124 with the complete EcoR124I system. For screening potential r^−^ mutants, “spot tests” were used as described previously.^42^ Individual r^−^ clones were analysed for modification function, and those with an r^−^m^+^ phenotype were conjugated into JM109(DE3) and analysed using a complementation assay. The restriction-deficient phenotype of JM109(DE3)[R124–25] was positively complemented by a WT *hsdR* gene introduced on pACR124.

### *In vitro* DNA cleavage assay

As a substrate for DNA cleavage *in vitro*, one-site plasmids pCFD30^43^ and pMDS27.3^27^ were used. Nuclease activity was analysed at either 37 ± 0.1 or 25 ± 0.1 °C in buffer R (50 mM Tris–Cl, pH 8.0, 10 mM MgCl_2_ and 1 mM DTT) using 5 nM DNA, 40 nM MTase and ≥ 100 nM HsdR. Following a brief preincubation (∼ 5 min), reactions were started by addition of ATP to a final concentration of 4 mM, and aliquots were quenched at the indicated times by addition of 0.5 volumes of STEB [0.1 M Tris–Cl, pH 8.0, 0.2 M ethylenediaminetetraacetic acid, 40% (w/v) sucrose and 0.4 mg/ml of bromophenol blue]. The covalently closed circular DNA substrate (CCC), open circle/nicked intermediate (OC) and full-length linear product (FLL) were separated by agarose gel electrophoresis, and the percentage of DNA in each band was evaluated by scintillation counting or by densitometry of the ethidium bromide fluorescence.

We analysed the apparent rate of CCC cleavage using the following formula:^8^

(1) $$y=A\left(1-{\text{e}}^{-k_{\text{cut,app}}\left(t-\text{offset}\right)}\right)$$

where *y* represents the appearance of the cleaved DNA, *A* is the percentage of DNA cut, *k*_cut,app_ is the apparent cleavage rate and “offset” is a time lag that represents the continuum of initiation/translocation/collision states prior to cleavage. The *y* values were calculated by subtracting the CCC concentration at each time point from the starting concentration and normalising the result to a 100% scale. The data that were not part of the lag phase were fitted to Eq. (3) with *A*, *k*_cut,app_ and “offset” allowed to float.

### Magnetic tweezers measurements

Magnetic tweezers experiments using 2.8-μm magnetic beads (Dynal) were carried out in buffer R supplemented with 4 mM ATP as described previously.^21^ The DNA substrate was prepared as described previously^21^ by cutting plasmid pSFV1 with SpeI and BamHI, providing a 10.2-kbp fragment with a single EcoR124I site 2.4 kbp from the BamHI site. The fragment was ligated to 600-bp-long biotinylated and digoxigenated PCR fragments cut with BamHI and SpeI, respectively, to form the final construct. The MTase concentration was 20 nM, the HsdR concentration was 160 nM and the applied stretching force was 1.5 pN. All measurements were carried out at 25 ± 2 °C. Data analysis was carried out as previously described.^19,21^

### Triple-helix displacement assay

Triplex displacement measurements were carried out in an SF61-DX2 stopped-flow fluorimeter as described previously.^19,20,27^ Reactions were initiated by mixing equal volumes of a DNA and enzyme solution with an ATP solution to give final conditions of 1 nM linear DNA (plus 0.5 nM tetramethylrhodamine TFO), 30 nM MTase, 120 nM HsdR, 100 μM AdoMet and 4 mM ATP in buffer R at 25 ± 0.1 °C. Lag times were estimated by fitting the profiles to a triple-exponential relationship:

(2) $$y=A_{1}\left(1-{\text{e}}^{-k_{1}(t-T_{\text{app}})}\right)+A_{2}\left(1-{\text{e}}^{-k_{2}(t-T_{\text{app}})}\right)+A_{3}\left(1-{\text{e}}^{-k_{3}(t-T_{\text{app}})}\right)$$

where *k*_*n*_ and *A*_*n*_ are the rate and amplitude, respectively, of the *n*th phase and *T*_lag_ is the sum of all time constants for the initiation and translocation steps.^27^ *k*_step_ (the translocation speed) and *k*_ini,app_ (the initiation speed) were then determined from the linear relationship between *T*_lag_ and distance (*d*, bp):

(3) $$T_{\text{lag}}=\left[\left(\frac{1}{k_{\text{step}}}\right)d\right]+\left(\frac{1}{k_{\text{ini}}}\right)$$

*k*_step_ values were determined from reactions at each of four triplex spacings (315, 954, 1517 and 2093 bp) by using ApaI-linearised pLKS5.^44^

### ATPase assay

ATPase activity was measured by rapid mixing in the SF61-DX2 stopped-flow fluorimeter using the coumarin-labelled phosphate binding protein as described previously.^18,31^ Reactions were initiated by mixing equal volumes of a DNA plus enzyme solution with an ATP solution to give final conditions of 0.2 nM ApaI-linearised DNA (pLKS5 or pTYB11), 60 nM MTase, 200 nM HsdR, 0 − 1200 μM ATP, 8 μM phosphate binding protein, 0.01 U/ml of bacterial purine nucleoside phosphorylase, 0.2 mM 7-methyl-guanosine and buffer R at 25 ± 0.1 °C. The response of the fluorimeter was calibrated using titration of a P_i_ standard^18^ and was shown to be linear across the range measured (data not shown). Linear steady-state phases of the ATPase rate profiles were fitted to:^18^

(4) $$C_{\text{p}}=\frac{1}{k_{\text{ATP}}}\cdot t+\tau_{\text{ini}}$$

where *C*_p_ is the phosphate concentration, *k*_ATP_ is the macroscopic steady-state ATPase rate, *τ*_ini_ is the macroscopic initiation delay time and *t* is the reaction time. Rates obtained on specific one-site DNA (linearised pLKS5) were corrected for background HsdR ATPase activity by subtracting rates determined in parallel on the non-specific DNA (linearised pTYB11). (pLKS5 and pTYB11 differ by only ∼ 2%.) The ATP-dependent rates obtained were fitted to a Michaelis–Menten relationship:

(5) $$v=\frac{V_{\max}[\text{ATP}]}{K_{\text{m}}+[\text{ATP}]}.$$

## Figures and Tables

**Table 1:** Effect on the restriction phenotype of changes in PD-(E/D)xK motifs II and III of the nuclease domain of EcoR124I HsdR

HsdR	Restriction^a^	
	r^−^ host^b^	r^+^ host^c^
WT	0.001	0.002
D151A	0.1	0.9
E165A	0.1	0.2
E165D	0.3	0.6
E165H	0.2	1.0
K167A	0.1	1.0

The *in vivo* restriction activity was quantified as the efficiency of plating, the ratio of the phage titre on the test host to the titre on a non-restricting host. Shown are the outcomes of our analysis of positive complementation in r^−^ host and negative complementation (transdominant effect) in r^+^ host.

a The efficiency of plating of λ*vir*.0 on tested strains relative to the efficiency of plating of λ*vir*.0 on *E. coli* C122 indicator (non-restricting) strain.

b *E. coli* JM109(DE3)[R124–25] (r^−^m^+^).

c *E. coli* JM109(DE3)[R124] (r^+^m^+^).

**Table 2:** Kinetic constants for WT and mutant HsdR subunits

	DNA cleavage			DNA translocation velocity			DNA translocation lifetime		ATPase		
				Tweezers		Bulk					
	Lag offset (s) (±SE)	Relative first first-strand rate^a^	*A* (%)	R_1__,slow_ (bp s^− 1^) (±SE)	R_1_ (bp s^− 1^) ( ±SE)	R_1_ (bp s^− 1^)^b^ ( ± SE)	R_1_ 〈*T*_event_〉(s)^c^	R_2_ 〈*T*_event_〉 (s)	*K*_m_ (μM) (±SE)	*V*_max_ (s^− 1^)^d^ (±SE)	Relative ATPase rate^e^
WT	10.1 ± 1.6	1	88	−	588 ± 6	599 ± 5	0.95 ± 0.18	3.23 ± 0.22	64 ± 4^f^	807 ± 11^f^	1.00
D151A	−	−	−	243 ± 45	490 ± 9	528 ± 4	2.75 ± 0.39	5.02 ± 0.57	50 ± 5	265 ± 15	0.32
E165A	88.6 ± 31.7	0.07	6	130 ± 5	320 ± 3	430 ± 22	2.33 ± 0.18	3.64 ± 0.16	58 ± 3	251 ± 9	0.31
E165D	59.0 ± 6.6	0.23	43	−	498 ± 3	611 ± 12	1.26 ± 0.20	3.42 ± 0.20	56 ± 3	333 ± 13	0.41
E165H	−	−	−	184 ± 3	370 ± 8	524 ± 8	2.10 ± 0.19	4.32 ± 0.33	43 ± 4	200 ± 12	0.25
K167A	−	−	−	345 ± 11	577 ± 5	608 ± 13	1.27 ± 0.16	2.24 ± 0.19	54 ± 5	503 ± 28	0.63

a *k*_cut,app,mutant_ relative to *k*_cut,app,WT_.

b Rate of one HsdR in an R_2_ complex (HsdR saturating) measured from triplex displacement.

c Mean lifetime for all R_1_ events, regardless of speed.

d ATP per HsdR assuming a fully saturated system and 100% occupancy.

e *V*_max,mutant_ relative to *V*_max,WT_.

f Data from Ref. 18. SE indicates standard error from fitted data.
